# Polypharmacy and self-medication among older adults in Indian urban communities—a cross-sectional study

**DOI:** 10.1038/s41598-024-84627-2

**Published:** 2025-02-03

**Authors:** Saibal Das, Pavithra Gnanavel, Shalini Smanla, Anku Moni Saikia, Shilpi Mishra, Shweta Khare, S. Arun Murugan, Vadanere Nidhi Prakash, Parimita Roychoudhury, Ishteyaque Ahmad, Vishal Diwan, J. Rajesh, K. Sathish Kumar, Jugal Kishore, Namita Srivastava, Sabrina Yasmin, Mahmuda Nasrin, Rinku Borah, Mandeep Sarma Basistha, Chetanjit Baruah, Manoj Kalita, Shambo Samrat Samajdar, Jerin Jose Cherian, Ashish Pathak, Samiran Panda, Santanu Kumar Tripathi, Cecilia Stålsby Lundborg

**Affiliations:** 1 Department of Global Public Health, Karolinska Institutet, Stockholm, Sweden; 2 Indian Council of Medical Research - Centre for Aging and Mental Health, Kolkata, India; 3 Department of Community Medicine, Government Medical College, Omandurar Government Estate, Chennai, India; 4 Department of Community Medicine, Vardhman Mahavir Medical College and Safdarjung Hospital, New Delhi, India; 5 Department of Community Medicine, Dhubri Medical College and Hospital, Dhubri, India; 6 Department of Pharmacology, Netaji Subhas Medical College and Hospital, Patna, India; 7 Department of Public Health Sciences and Environment, RD Gardi Medical College, Ujjain, India; 8 Indian Council of Medical Research - National Institute for Research in Environmental Health, Bhopal, India; 9 Department of Community Medicine, Kilpauk Medical College, Chennai, India; 10 Department of Pharmacology, Nagaon Medical College Hospital, Nagaon, India; 11 Department of Community Medicine, Tinsukia Medical College and Hospital, Tinsukia, India; 12 Department of Pharmacology, JNM Medical College and Hospital, Nadia, India; 13 Indian Council of Medical Research, New Delhi, India; 14 Department of Pediatrics, RD Gardi Medical College, Ujjain, India; 15 Jagannath Gupta Institute of Medical Sciences and Hospital, Kolkata, India

**Keywords:** Older adults, Polypharmacy, Potentially inappropriate medications (PIMs), Potential prescribing omissions (PPOs), Self-medication, Screening tool of older persons’ prescriptions and screening tool to alert to right treatment (STOPP-START), Health care, Medical research

## Abstract

Older adults are vulnerable to unsafe medication practices. This cross-sectional study estimated the prevalence and factors of polypharmacy and self-medication among 600 older adults from six Indian cities. The updated Screening Tool of Older Persons’ Prescriptions and Screening Tool to Alert to Right Treatment criteria (version 3) were used. Knowledge, attitudes, and reported practices regarding self-medication were assessed. Descriptive statistics, binary logistic regression, and multivariable analysis were used. The prevalence of polypharmacy was 33.7% (95% CI 29.9–37.6%), with significant associations to multiple comorbidities [adjusted odds ratio (aOR) 2.5 (95% CI 1.1–4.1)], recent transition of care [aOR 3.3 (95% CI 1.4–5.7)], and recent hospitalization [aOR 4.6 (95% CI 2–7.7)]. The proportions of prescriptions with potentially inappropriate medications and potential prescribing omissions were 28.8% (95% CI 25.2–32.6%) and 20.3% (95% CI 17.2–23.8%), respectively. The prevalence of self-medication was 19.7% (95% CI 16.6–23.1%), associated with factors, such as staying alone [aOR 4.5 (95% CI 2.4–6.6)], multiple comorbidities [aOR 3 (95% CI 1.4–6.7)], and recent hospitalization [aOR 4.8 (95% CI 1.5–8)]. Among those who self-medicated, 65.3% lacked knowledge of self-medication, 50% did not comprehend the risks, and 40.7% reported unsafe self-medication practices. The findings emphasize interventions’ necessity for promoting safe medication use in older adults.

## Introduction

Presently, the proportion of persons aged ≥ 60 years (defined by the United Nations), hereafter referred to as ‘older adults’, in India is 10.1%^1^. Older adults are often vulnerable to multiple comorbidities and social constraints and require special attention. With advancements in medical science, the lifespan has increased, and the proportion of older adults is expected to double by 2050^1^. However, geriatric health is sometimes not prioritized in India^2^. Along with age-related diseases, older adults are often exposed to the risk of inappropriate medication use, particularly when exposed to polypharmacy and uninformed and unsafe self-medication, which can increase the likelihood of harm if not carefully monitored^3^.

Polypharmacy is defined by the World Health Organization (WHO) as “the administration of many drugs at the same time or the administration of an excessive number of drugs”^4^. Despite several heterogeneities and the absence of a unanimous contextual definition, the most common working definition refers to a numerical intake of five or more medications per day^5^. Polypharmacy increases the risk of potentially inappropriate medications (PIMs), which need to be de-prescribed, and potential prescribing omissions (PPOs), i.e., medicines that are missed in the prescription^6^. Older adults have altered physiological status and sometimes multiple comorbidities altering the pharmacokinetics and pharmacodynamics of medicines. Polypharmacy is closely related to comorbidity. It can be necessary and rational when each medication is prescribed with a clear clinical indication, helping to effectively manage comorbidities and improve health outcomes^4^. On the other hand, inappropriate polypharmacy occurs when one or more medications are prescribed unnecessarily due to a lack of evidence-based indication, failure to achieve therapeutic goals, absence of medication reconciliation support when multiple physicians are involved in treating a patient, or when the patient adherence is low^4^. Reduced medication adherence in the elderly is often influenced by factors, such as cognitive deficits, complex medication regimens, adverse effects, financial constraints, and a lack of understanding or awareness about the importance of following prescribed treatments^7^. Hence, it is important to assess prescribing behavior, as well as medications taken by older adults, for their appropriateness.

The other common type of inappropriate medication practice among older adults is uninformed and unsafe self-medication, which is defined as the use of medications without a legitimate prescription by a registered medical practitioner^8^. Because of frailty, multiple comorbidities, and practical challenges in accessing authorized healthcare facilities, older adults are often exposed to unsupervised self-medication, sometimes with traditional and complementary medicines^9^. Such self-medication practices are induced not only by oneself but also by family members, friends, or other personnel^10^. In addition to posing a risk to the individual (e.g., incorrect diagnosis, drug-drug interactions, adverse drug reactions), inappropriate self-medication also poses a risk to the community (e.g., antimicrobial resistance)^11^. The problem is more pronounced in this population than in the general population in India compared to Western countries because of the absence of guidelines for licensing over-the-counter medicines, the absence of structured medication reconciliation support, and the unregulated dispensing of medicines without proper diagnosis by unqualified practitioners^12,13^.

The burden and risk factors associated with polypharmacy and self-medication in older adults vary across countries. Given the global focus of the WHO on patient safety^14^ and medication without harm^15^, it is important to identify unsafe medication practices that can potentially harm individual patients and/or society. Such practices can be avoided to mitigate this problem. This is very relevant for older adults. In India, cultural variations and low levels of health awareness influence medication practices across different communities^16^. Furthermore, well-established and authorized geriatric care facilities for individuals are not yet available across the country. Irrational polypharmacy and self-medication have been reported to be serious healthcare issues in India, especially in the urban population^17,18^. This problem appears to have been aggravated following the coronavirus disease 2019 (COVID-19) pandemic^19^. Although some studies on polypharmacy and self-medication in older adults have been conducted in hospital settings in India, community-based studies are sparse^18,20^. Additionally, the associated factors were not elucidated in detail. The burden and factors associated with unsafe medication practices in older adults in Indian community settings might be different from those reported in hospital settings. Hence, this study was performed to estimate the prevalence and factors associated with polypharmacy and self-medication among older adults residing in six major Indian cities located in different geographical regions.

## Methods

### Study design and setting

This cross-sectional study was conducted in selected urban communities^21^ from six different geographical regions [New Delhi (northern part): Vardhman Mahavir Medical College and Safdarjung Hospital; Chennai (southern part): Government Medical College, Omandurar Government Estate; Kolkata (eastern part): Indian Council of Medical Research—Centre for Aging and Mental Health; Ujjain (central part): RD Gardi Medical College; Patna (eastern part): Netaji Subhas Medical College and Hospital; and Guwahati (northeastern part): Gauhati Medical College and Hospital].

### Study population

A line listing of houses with older adults (aged ≥ 60 years) in the respective urban field practice areas for health research at each study site (medical colleges and research centers) in each city was available. Then, the households were selected and included by systematic random sampling until the sample size was achieved. In households where there was more than one older adult individual, only the eldest one was included. Individuals who were incapable of independent daily living and those with severe cognitive or hearing deficits (identified through caregiver reports on memory loss, decrease in daily functioning, confusion, declining language skills, and alterations in behavior; as well as basic patient evaluations during the initial screening process) that prevented comprehension and participation were excluded.

### Data collection

Door-to-door household surveys using a pretested questionnaire in local languages were conducted between November 2022 and January 2024 until the sample size was reached. The interviewers, with backgrounds in medicine, pharmacy, or public health received comprehensive training led by senior researchers and clinicians prior to data collection. The sociodemographic characteristics and medical history of the individuals were recorded. The details of all present allopathic medications taken within the preceding three months were collected and obtained from prescriptions, medical records, medication strips/bottles, pharmacy bills, etc., and from interviews with individuals and/or caregivers. The details of the present intake of traditional and complementary medications were also recorded. Medical records were referred to by asking older adult individuals or their caregivers to provide any available documentation. The operational definition of polypharmacy was the present intake of five or more daily allopathic medications (solid oral formulations) for at least four weeks in the last three months^5^. The details of other oral allopathic medications (liquid formulations) and medications taken through nonoral routes were also recorded. The updated STOPP-START (Screening Tool of Older Persons’ Prescriptions and Screening Tool to Alert to Right Treatment) criteria (version 3) were used to identify PIMs and PPOs, respectively^22^ by qualified Clinical Pharmacologists. The operational definition of self-medication was the use of over-the-counter and prescription medicines without the legitimate prescription of a registered medical practitioner or the use of a prescribed medicine without adhering to the instructions of a registered medical practitioner for the present illness^8,23^. The knowledge, attitudes, and self-reported practices of the individuals regarding self-medication were assessed using a questionnaire that was pre-validated among college students in an earlier Indian study^24^. The questionnaire was translated from English into the regional languages of each city (Bengali, Tamil, Hindi, and Assamese) following a standardized protocol with forward and backward translation^4^. Bilingual experts reviewed each version to ensure consistency, address discrepancies, and maintain cultural relevance. The fourteen questions on knowledge were related to the awareness and understanding of responsible self-medication, including knowledge of medicine actions, reasons for continuation or discontinuation, proper usage, potential harm, storage, timing, adverse effects, dosage, actions for adverse effects, and information resources. The seven questions on attitudes were related to risks, unsafe dosages, long-term use, adverse effects, and professional advice. The five questions on reported practices were related to reading package inserts, sharing medicines, taking professional advice, types of diseases, and practicing self-medication for a long time. Clinical pharmacologists and geriatricians were consulted to review each item of the questions for relevance and clarity (face validity). Based on their recommendations, the language was simplified, and certain items were modified to better align with older adults in India.

### Outcomes

The primary outcome was the prevalence of polypharmacy and the prevalence of polypharmacy self-medication. The secondary outcomes were the spectrum of polypharmacy; the spectrum of PIMs and PPOs (among all recruited individuals); the spectrum of self-medication; factors associated with polypharmacy and self-medication; and knowledge, attitudes, and reported practices regarding self-medication.

### Sample size calculation

Assuming a polypharmacy prevalence of 50%^18^ and a self-medication prevalence of 54%^10^ in older adults, the estimated sample size was 601 (~ 600) at a 4% absolute precision level and a 5% standard normal deviation.

### Statistical analysis

Descriptive statistics were used for the sociodemographic characteristics, the prevalence of polypharmacy (including PIMs and PPOs), and the prevalence of self-medication. Binary logistic regression was used to identify potential risk factors associated with polypharmacy and self-medication. In the first step, a univariate analysis was performed. The factors included were age, sex, marital status, number of members staying in the same house, education level of the individual, education level of the primary (at the time of the study) caregiver (family member or friend or nurse who provides service for daily care of the older adult), occupation, per capita monthly income, comorbidities, transition of care (the movement of a patient from one setting of care to another) within the preceding three months, and hospitalization within the preceding three months. In the second step, factors that were found to be significant in the univariate analysis (p-value < 0.2) were included in the multivariable model. The study employed the enter method to conduct a multivariable analysis of risk factors, aiming to construct a model while also controlling for confounding variables. For knowledge, attitudes, and reported practices, descriptive statistics were calculated using frequency distributions for each component. A p-value of < 0.05 was considered to indicate statistical significance in the final model. All analyses were conducted using SPSS version 23 (IBM, Armonk, NY).

### Ethics

The study was conducted following the Declaration of Helsinki and ICMR-National Ethical Guidelines for Biomedical and Health Research Involving Human Participants, 2017. Approval was obtained from the appropriate ethics committee(s) of the individual study sites (Indian Council of Medical Research—Centre for Aging and Mental Health, Kolkata; Government Medical College, Omandurar Government Estate, Chennai; Vardhman Mahavir Medical College and Safdarjung Hospital, New Delhi; Dhubri Medical College and Hospital, Dhubri, India; Netaji Subhas Medical College and Hospital, Patna; and RD Gardi Medical College, Ujjain). Informed consent was obtained from individuals/family members before recruitment and interviews. When serious issues related to medications were identified, individuals were advised to seek guidance from their healthcare professionals for further evaluation and adjustment of their medication regimens.

## Results

A total of 100 older adults were recruited in each city (*n* = 600). The mean ± standard deviation age of the individuals was 72 ± 3.6 years, and 58.8% of the individuals were females. The sociodemographic characteristics of the study population are enumerated in Table 1. Multiple (three or more) comorbidities were present among 423 (70.5%) individuals. A total of 131 (21.8%) individuals had transitioned to care within the preceding three months, and 71 (11.8%) individuals had a history of hospitalization within the preceding three months.

**Table 1:** Sociodemographic characteristics of the study population (*n* = 600).

Sociodemographic characteristics		Number (%)
Age (years)	60–80	477 (79.5)
	> 80	123 (20.5)
Sex	Female	353 (58.8)
Marital status	Never married	13 (2.2)
	Married	476 (79.3)
	Divorced/separated	14 (2.3)
	Widow	97 (16.2)
Number of members staying in the same house	One	25 (4.2)
	Two to five	480 (80)
	More than five	95 (15.8)
Educational level of the individual	Primary school	86 (14.3)
	Secondary school	401 (66.8)
	Graduate and above	113 (18.9)
Educational level of the primary caregiver	Primary school	113 (18.9)
	Secondary school	459 (76.4)
	Graduate and above	28 (4.7)
Occupation of the individual	Retired	476 (79.3)
	Worker	45 (7.5)
	Business	43 (7.2)
	Service/consultant	36 (6)
Per capita monthly income of the family (Indian Rupees) (1 Euro = 90.58 Indian Rupees as of 15 May 2024)	< 10,000	51 (8.5)
	10,000–25,000	534 (89)
	> 25,000	15 (2.5)
Documented present medical illnesses (comorbidities)	Hypertension	392 (65.3)
	Gastroesophageal reflux disease and acid peptic disorder	367 (61.2)
	Type 2 diabetes mellitus	356 (59.3)
	Hyperlipidemia	302 (50.3)
	Osteoporosis	300 (50)
	Cataract	266 (44.3)
	Rheumatic diseases	234 (39)
	Coronary artery disease	180 (30)
	Congestive heart failure	112 (18.7)
	Stroke	112 (18.7)
	Chronic obstructive pulmonary disease and asthma	108 (18)
	Alzheimer’s disease	81 (13.5)
	Chronic kidney disease	56 (9.3)
	Parkinson’s disease	54 (9)
	Gynecological diseases	45 (7.5)
	Cancer	28 (4.7)
Number of comorbidities	One to two	177 (29.5)
	Three or more	423 (70.5)
Transition of care within the preceding three months	Yes	131 (21.8)
	No	469 (78.2)
Hospitalization within the preceding three months	Yes	71 (11.8)
	No	529 (88.2)

The overall prevalence of polypharmacy was 33.7% [95% confidence interval (CI) 29.9–37.6%]. A total of 84 (12%) of the individuals were not taking any medicine, and 314 (54.3%) individuals were taking one to four oral allopathic medications (solid formulations) daily. A total of 88 (14.7%) individuals were taking oral allopathic medications (liquid formulations), and 71 (11.8%) of the individuals were taking allopathic medications through non-oral routes daily. Among the participating individuals, the highest prevalence of polypharmacy was found in Guwahati (57%), while the lowest prevalence was in Ujjain (11.7%). A total of 2,741 medicines (solid oral formulations) were prescribed to all individuals; the most common were antihypertensive medicines (19.9%), followed by antidiabetic medicines (15.9%), hypolipidemic medicines (13.9%), calcium supplements (13%), and nonsteroidal anti-inflammatory drugs (13%). As many as 25.2% (95% CI 21.7–28.4%) of the individuals used at least one fixed-drug combination (solid oral formulations); the most common combinations were antihypertensive and antidiabetic fixed-drug combinations. A total of 164 (27.3%) individuals were using concomitant traditional and complementary medications (Ayurveda, Unani, Siddha, and Homeopathy), mostly for chronic conditions. Among these individuals, 80 (48.8%) used certain traditional and complementary solid oral formulations without names or labels. The statistically significant factors that were associated with polypharmacy were the presence of three or more comorbidities [adjusted odds ratio (aOR) 2.5 (95% CI 1.1–4.1), *p* = 0.03], recent transition of care within 3 months [aOR 3.3 (95% CI 1.4–5.7), *p* = 0.02], and recent hospitalization within 3 months [aOR 4.6 (95% CI 2-7.7), *p* < 0.001] (Table 2).

**Table 2:** Factors associated with polypharmacy and self-medication (*n* = 600).

Factors associated with polypharmacy			
Factors included in the univariate analysis		Unadjusted odds ratio (95% CI)	p-value
Age		1.4 (0.9–2.9)	0.23
Sex	Female	1	
	Male	2 (0.9–3.5)	0.25
Marital status	Never married	1	
	Married	1.6 (0.8–3.6)	0.19
Number of members staying in the same house	More than one	1	
	One	1.6 (0.8–2.9)	0.15
Education level of the individual	Secondary school	1	
	Below secondary school	4.1 (0.7–7.4)	0.08
Education level of the primary caregiver	Secondary school	1	
	Below secondary school	1.5 (0.5–3.1)	0.12
Occupation	Presently working	1	
	Presently not working or retired	1.6 (0.7–3.8)	0.19
Per capita monthly income	≥Indian Rupees 10,000	1	
	< Indian Rupees 10,000	3.3 (0.1–5.9)	0.19
Comorbidities	One or two	1	
	Three or more	2.7 (1.3–4.9)	0.03
Transition of care within the preceding three months	Yes	1	
	No	3.1 (1.6-6)	0.02
Hospitalization within the preceding three months	Yes	1	
	No	4.3 (2.2–7.5)	< 0.001

Factors included in the multivariable analysis		Adjusted odds ratio (95% CI)	p-value
Marital status	Never married	1	
	Married	1.8 (0.6–3.3)	0.21
Number of members staying in the same house	More than one	1	
	One	1.4 (0.6–2.7)	0.15
Education level of the individual	Secondary school	1	
	Below secondary school	3.9 (0.8–6.9)	0.31
Education level of the primary caregiver	Secondary school	1	
	Below secondary school	1.1 (0.3–2.7)	0.12
Occupation	Presently working	1	
	Presently not working or retired	1.8 (0.5–3.3)	0.21
Per capita monthly income	≥Indian Rupees 10,000	1	
	< Indian Rupees 10,000	2.9 (0.9–5.1)	0.06
Comorbidities	One or two		
	Three or more	2.5 (1.1–4.1)	0.03
Transition of care within the preceding three months	Yes	1	
	No	3.3 (1.4–5.7)	0.02
Hospitalization within the preceding three months	Yes	1	
	No	4.6 (2-7.7)	< 0.001

Factors associated with self-medication			
Factors included in the univariate analysis		Unadjusted odds ratio (95% CI)	p-value
Age		1.6 (0.9–4.2)	0.22
Sex	Female	1	
	Male	1.2 (0.1–4.1)	0.26
Marital status	Never married	1	
	Married	1.8 (0.7–6.1)	0.15
Number of members staying in the same house	More than one	1	
	One	4.2 (2.1-7)	< 0.001
Education level of the individual	Secondary school	1	
	Below secondary school	3.6 (0.6–7.9)	0.06
Education level of the primary caregiver	Secondary school	1	
	Below secondary school	1.7 (0.9–3.7)	0.14
Occupation	Presently working	1	
	Presently not working or retired	2.2 (0.8–4.6)	0.07
Per capita monthly income	≥Indian Rupees 10,000	1	
	< Indian Rupees 10,000	2.3 (0.8–6.1)	0.17
Comorbidities	One or two	1	
	Three or more	3.2 (1.5–6.9)	0.03
Transition of care within the preceding three months	Yes	1	
	No	4.8 (0.8–6.8)	0.18
Hospitalization within the preceding three months	Yes	1	
	No	5 (1.4–8.4)	0.04

Factors included in the multivariable analysis		Adjusted odds ratio (95% CI)	p-value
Marital status	Never married	1	
	Married	1.9 (0.6–6.6)	0.16
Number of members staying in the same house	More than one	1	
	One	4.5 (2.4–6.6)	< 0.001
Education level of the individual	Secondary school	1	
	Below secondary school	3.5 (0.9–8.2)	0.08
Education level of the primary caregiver	Secondary school	1	
	Below secondary school	1.8 (0.8–3.8)	0.06
Occupation	Presently working	1	
	Presently not working or retired	2.4 (0.7–4.8)	0.17
Per capita monthly income	≥Indian Rupees 10,000	1	
	< Indian Rupees 10,000	2.4 (0.7–6.2)	0.16
Comorbidities	One or two		
	Three or more	3 (1.4–6.7)	0.01
Transition of care within the preceding three months	Yes	1	
	No	5.1 (0.9–6.9)	0.17
Hospitalization within the preceding three months	Yes	1	
	No	4.8 (1.5-8)	0.04

*CI* confidence interval.

Among all recruited individuals, based on the STOPP criteria, 173 [28.8% (95% CI 25.2–32.6%)] were prescribed at least one PIM. The highest occurrence of PIM use, at 46.2%, pertained to prescriptions lacking evidence-based clinical justification, medications prescribed beyond the recommended duration, and instances of duplicate therapy. The most commonly identified PIMs included long-term use of benzodiazepines (69.4%), prolonged use of proton pump inhibitors without appropriate indications (56.6%), nonsteroidal anti-inflammatory drugs without gastroprotection (37.6%), and duplicate therapy with medications from the same class (35.8%). According to the START criteria, among all recruited individuals, 122 [20.3% (95% CI 17.2–23.8%)] had at least one PPO. The details of the PIMS and PPOs according to the STOPP-START criteria are listed in Table 3. The prevalent PPOs among these individuals included antiplatelet therapy for diabetes mellitus if one or more major cardiovascular risk factors were present (39.3%), statin therapy for diabetes mellitus if one or more major cardiovascular risk factors were present (34.4%), aspirin or clopidogrel for individuals with documented atherosclerotic coronary, cerebral, or peripheral vascular disease and sinus rhythm (32.8%), calcium and vitamin D supplementation for individuals with known osteoporosis (31.1%), and metformin for individuals with type 2 diabetes with or without metabolic syndrome (26.2%). There were no documented contraindications for the use of these medications.

**Table 3:** Details of potentially inappropriate medications (PIMs) and potential prescribing omissions (PPOs) as per the screening tool of older persons’ prescriptions and screening tool to alert to right treatment (STOPP-START) criteria.

	*n* (%)	95% CI
PIM as per the STOPP criteria		
Individuals prescribed with at least one PIM (*n* = 600)	173 (28.8)	25.2–32.6
Individuals prescribed with one PIM (*n* = 173)	100 (57.8)	50.1–65.3
Individuals prescribed with two PIMs (*n* = 173)	41 (23.7)	17.6–30.8
Individuals prescribed with three PIMs (*n* = 173)	23 (13.3)	8.6–19.3
Individuals prescribed with four or more PIMs (*n* = 173)	9 (5.2)	2.4–9.7
PPO as per the START criteria		
Individuals having with at least one PPO (*n* = 600)	122 (20.3)	17.2–23.8
Prescription with one PPO (*n* = 122)	68 (55.7)	46.5–64.7
Prescription with two PPOs (*n* = 122)	32 (26.2)	18.7–34.9
Prescription with three PPOs (*n* = 122)	15 (12.3)	7.1–19.5
Prescription with four or more PPOs (*n* = 122)	7 (5.8)	2.3–11.5

*CI* confidence interval, *PIMS* potentially inappropriate medications, *PPOs* potential prescribing omissions, *STOPP-START* screening tool for older persons’ prescriptions and screening tool for identifying the right treatment.

The overall prevalence of self-medication (over-the-counter and prescription medicines) was 19.7% (95% CI 16.6–23.1%). Among the participating individuals, the highest prevalence of self-medication was found in Kolkata (38.3%), while the lowest prevalence was in Ujjain (4%). The most common medicines that were self-medicated were nonsteroidal anti-inflammatory drugs (59%), followed by paracetamol (42.4%), and antibiotics for upper respiratory tract infection and diarrhea (33.9%). The statistically significant factors that were associated with self-medication were remaining alone [aOR: 4.5 (95% CI 2.4–6.6), *p* < 0.001], the presence of three or more comorbidities [aOR 3 (95% CI 1.4–6.7), *p* = 0.01], and recent hospitalization within 3 months [aOR 4.8 (95% CI 1.5-8), *p* = 0.04] (Table 2). Among those who self-medicated (118, 19.7%), 65.3% lacked knowledge of uninformed or unsafe self-medication, 50% did not comprehend the associated risks, and 40.7% reported unsafe self-medication practices (Table 4).

**Table 4:** Knowledge, attitudes, and reported practices regarding self-medication among those who were found to self-medicate (*n* = 118).

Domain		Number (%)
Knowledge		
Awareness about responsible selfmedication	Yes	41 (34.7)
	No	77 (65.3)
Basic knowledge about medicine action for self-medication	Yes	38 (32.2)
	No	80 (67.8)
Awareness about the discontinuation of medicines preferred for selfmedication	Yes	30 (25.4)
	No	83 (70.3)
	Do not know	5 (4.3)
Aware of reasons to continue or discontinue medications preferred for selfmedication	Yes	40 (33.9)
	No	78 (66.1)
Medicines should be continued even after symptoms are relieved up to the course of the regimen	Yes	26 (22)
	No	79 (66.9)
	Do not know	13 (11.1)
Names of illnesses where selfmedication will be recommended	Fever	86 (72.8)
	Cough and cold	87 (73.7)
	Headache	68 (57.6)
	Body ache	47 (39.8)
	Diarrhea	71 (60.2)
	Vomiting	47 (39.8)
	Gastritis	67 (56.8)
	Others	33 (27.9)
Medicines that are preferred for selfmedication do not cause any harm even in higher doses	Yes	46 (38.9)
	No	39 (33.5)
	Do not know	33 (27.6)
Medicines taken as selfmedication can be stored at any temperature	Yes	59 (50)
	No	23 (19.5)
	Do not know	36 (30.5)
Selfmedication can be taken at any time of the day irrespective of food	Yes	54 (45.8)
	No	39 (33.1)
	Do not know	25 (21.1)
Knowledge about specific adverse effects of medicines preferred for selfmedication	Yes	24 (20.3)
	No	94 (79.7)
Knowledge course and dosage of medicines preferred for selfmedication	Yes	18 (15.3)
	No	100 (84.7)
During selfmedication if any unwanted effect appears, what will you do?	Discontinue	68 (57.6)
	Take another medication	25 (21.2)
	Consult doctor	21 (17.8)
	Others	4 (3.4)
Knowledge about information resources preferred for selfmedication	Previous experience	30 (25.4)
	Previous prescriptions	17 (14.4)
	Consulting family member or friend	18 (15.3)
	Consulting pharmacist	34 (28.8)
	Advertisement	4 (3.4)
	Books or newspapers	2 (1.7)
	Social media	1 (0.8)
	Others	12 (10.2)
Knowledge about reasons to favor selfmedication	Time-saving	47 (39.8)
	Economical	23 (19.5)
	Quick relief	20 (16.9)
	No need to visit a doctor	13 (11)
	Ease and convenience	8 (6.8)
	During emergency	4 (3.4)
	To avoid crowds during the Coronavirus Disease-19 pandemic	1 (0.8)
	Others	2 (1.8)
Attitude		
Selfmedication is not safe for older adults	Strongly agree	23 (19.5)
	Agree	16 (13.6)
	Neither agree nor disagree	9 (7.6)
	Disagree	59 (50)
	Strongly disagree	11 (9.3)
All dosage ranges of medicines preferred for selfmedication are not safe	Strongly agree	21 (17.8)
	Agree	28 (23.7)
	Neither agree nor disagree	25 (21.2)
	Disagree	20 (16.9)
	Strongly disagree	24 (20.4)
Selfmedication is not advisable for a prolonged period	Strongly agree	78 (66.1)
	Agree	26 (22)
	Neither agree nor disagree	7 (5.9)
	Disagree	2 (1.7)
	Strongly disagree	5 (4.3)
Close monitoring of symptoms and possible side effects is required during selfmedication	Strongly agree	23 (19.5)
	Agree	27 (22.9)
	Neither agree nor disagree	29 (24.6)
	Disagree	18 (15.3)
	Strongly disagree	21 (17.7)
Selfmedication is not completely free of adverse drug reactions	Strongly agree	23 (19.5)
	Agree	43 (36.4)
	Neither agree nor disagree	20 (16.9)
	Disagree	20 (16.9)
	Strongly disagree	12 (10.3)
Selfmedication medicines tend to interact with prescription medicines and food	Strongly agree	22 (18.6)
	Agree	29 (24.6)
	Neither agree nor disagree	33 (27.9)
	Disagree	16 (13.6)
	Strongly disagree	18 (15.3)
Selfmedication under professional advice will give better outcomes	Strongly agree	65 (55.1)
	Agree	28 (23.7)
	Neither agree nor disagree	15 (12.8)
	Disagree	5 (4.2)
	Strongly disagree	5 (4.2)
Reported practice		
Do you take selfmedication without reading the leaflet/package insert	Yes	70 (59.3)
	No	48 (40.7)
Have you given your prescription to someone who has similar symptoms	Yes	29 (24.6)
	No	89 (75.4)
Do you take medicines for selfmedication without any professional advice or prior knowledge	Yes	13 (11.1)
	No	105 (88.9)
Do you take medicines for selfmedication for long periods without any medical advice	Yes	18 (15.3)
	No	100 (84.7)
In all types of illnesses, do you prefer selfmedication	Yes	6 (5.1)
	No	112 (94.9)

## Discussion

The study, which was conducted across six Indian cities, revealed significant findings regarding medication usage and self-medication practices among older adults. Polypharmacy was prevalent, with 33.7% of individuals taking multiple medications. Traditional and complementary medicines were also common. Statistically significant factors associated with polypharmacy included comorbidities, recent transitions of care, and hospitalizations. Additionally, 28.8% were prescribed PIMs, while 20.3% had PPOs according to the STOPP-START criteria. Self-medication was prevalent at 19.7%, with notable associations with living alone, comorbidities, and recent hospitalizations. A lack of awareness and unsafe practices were found among self-medicating individuals. These findings underscore the complexity of medication management among older adults and highlight the need for comprehensive interventions to optimize medication use and promote safe practices.

This study provides a comprehensive overview of a diverse older adult population across six Indian cities from different geographical regions, highlighting key sociodemographic and health characteristics. The mean age of 72 years in our study population represented a predominantly older adult cohort, which aligns with the increasing prevalence of chronic conditions in older adults. The presence of three or more comorbidities among 70.5% of individuals underlines the complexity of managing healthcare needs in this population, necessitating a holistic approach to care delivery. At the same time, the disparity between the proportion of individuals having three or more comorbidities and the prevalence of polypharmacy possibly suggests undertreatment or poor treatment adherence. Furthermore, the findings indicate that a notable proportion of individuals experienced transitions of care and recent hospitalizations within the preceding three months, which could indicate potential gaps in continuity of care and indicate the importance of strategies to improve care coordination among healthcare providers and prevent adverse outcomes.

This study addresses the prevalence of polypharmacy among older adults, with 33.7% of individuals being prescribed multiple medications concurrently. The wide variation in the prevalence of polypharmacy across different cities ranging from 11 to 57% suggests potential regional differences in prescribing practices, healthcare-seeking behavior, or healthcare access, warranting further investigations. The analysis of prescribed medications revealed the predominant use of antihypertensive, antidiabetic, and hypolipidemic medications, reflecting the high burden of cardiovascular risk factors and chronic conditions within the population. Although appropriate polypharmacy is not harmful and can be rational^4^, our findings imply the complexity of managing healthcare in older adults, particularly those with chronic conditions, and emphasize the need for careful medication management to mitigate potential adverse effects and drug interactions. The contextuality of defining polypharmacy (e.g., from the perspective of medication adherence, drug-drug interactions, adverse drug reactions, cost, etc.) might be linked to its rationality^4^.

Polypharmacy is often associated with adverse outcomes, such as falls, frailty, disability, and mortality, in older adults, depending on the type of medicine included^25,26^. As in our study, the high burden of polypharmacy among older adults in India^27^ and other parts of the world^28–30^ has been previously reported in original studies and meta-analyses. A previous systematic review showed that the pooled prevalence of polypharmacy was 49% (95% CI 42–56%), and that of PIM use was 28% (95% CI 24–32%) among older adults^18^. A study in the Indian state of Karnataka reported a high prevalence of PIM use (54%) among older adults, and polypharmacy was found to be a risk factor for PIM use^31^. Another literature review noted that the Indian states of Uttaranchal, Karnataka, and Telangana reported a high prevalence of polypharmacy (93.14%, 84.6%, and 82.8%, respectively) among older adults^32^. A recent systematic review and meta-analysis including 94 articles and 371.2 million older adults from 17 countries reported an overall pooled prevalence of PIM use of 36.7%, and PIM use among older adults has become increasingly prevalent in the past two decades^33^.

The marked utilization of fixed-drug combinations and traditional complementary medications, as shown in our study, points toward the diversity of treatment approaches adopted by individuals, including both conventional and alternative therapies. However, the presence of unlabeled solid oral formulations among traditional medications raises concerns regarding patient safety and underlines the importance of regulatory oversight and adherence to quality standards in healthcare delivery. Furthermore, the identified associations between polypharmacy and factors, such as multiple comorbidities, recent transitions of care, and hospitalizations within three months highlight the interconnectedness between health status, healthcare utilization, and medication complexity^34^. Regrettably, evidence on the effectiveness and safety of medications in older adults dealing with multiple health conditions and taking multiple medications is scarce. This is primarily because such individuals are often not adequately represented or are excluded from clinical trials. Furthermore, clinical guidelines predominantly concentrate on single diseases and only occasionally touch upon aspects, such as care coordination, the timing and process of discontinuing treatments when necessary, or the prioritization of recommendations for those managing multiple health issues and medications^35^. According to the WHO, “Medication reviews in polypharmacy should take into account the effectiveness and the risk-benefit ratio of the medication treatment options, and examine these criteria for the specific patient group in which the medication is being used. Where possible, medication reviews should be performed in collaboration with the patient or their caregiver”^4^. Our findings stress the importance of comprehensive clinical pharmacological reconciliation, review, and feedback, particularly during care transitions, to optimize medication regimens and reduce the risk of adverse drug events^36^. Patient-centered deprescribing can be a good option in this regard^37^.

This study sheds light on PIM prescribing and PPO among older adults, as identified by the STOPP-START criteria. The findings reveal a concerning prevalence of PIM use, with 28.8% of individuals being prescribed at least one PIM. The highest prevalence of PIM use was attributed to medications prescribed without evidence-based clinical indications and those prescribed beyond the recommended duration. This underscores critical areas that require enhancement in prescribing practices. Additionally, the study identified common PIMs, such as duplicate therapy, underscoring the need for vigilant medication review and adherence to evidence-based guidelines to minimize the risk of adverse drug events in older adults. Conversely, the identification of PPOs through the START criteria highlights the importance of ensuring that individuals receive appropriate and evidence-based pharmacotherapy. According to the study findings, 20.3% of individuals exhibited at least one PPO. Common PPOs identified in the study included antiplatelet therapy for patients with diabetes mellitus having major cardiovascular risk factors, statin therapy for patients with diabetes mellitus having major cardiovascular risk factors, and calcium and vitamin D supplementation for individuals diagnosed with osteoporosis. Similar to our findings, earlier studies have also shown the high burden of inappropriate prescriptions (PIMs and PPOs) among older adults^4^. On the one hand, PIM is significantly associated with a range of health-related and system‐related outcomes in older adults^38^, and on the other hand, chronic diseases and advanced age are significantly associated with PIM use^39^. These findings emphasize the importance of proactive management of chronic conditions and adherence to guideline-recommended pharmacotherapy to optimize patient outcomes and reduce the risk of disease progression and complications^40^.

This study sheds light on the prevalence and characteristics of self-medication practices among the patient population, revealing important implications for healthcare delivery and patient safety. With an overall prevalence of 19.7%, self-medication has emerged as a common phenomenon, particularly in certain regions, such as Kolkata, where it reached 38%. Notably, in our operational definition of self-medication, we included both over-the-counter and Schedule H and H1 (prescription) medicines^41^. Nonsteroidal anti-inflammatory drugs, paracetamol, and antibiotics are among the most frequently used self-medicated medications, reflecting a reliance on self-care practices for managing common ailments, such as pain and infections. Although the short-term use of nonsteroidal anti-inflammatory drugs and paracetamol for self-medication can be acceptable, the use of antibiotics without the advice of any registered medical practitioner may be inappropriate. The identified factors associated with self-medication, such as living alone, multiple comorbidities, and recent hospitalization, underscore the multifaceted nature of self-care behaviors influenced by individual circumstances and healthcare experiences. An earlier study showed that having a low education level, being single, and staying alone significantly reduced the quality of life among urban-dwelling older adults^42^. Studies on self-medication in older adults and its adverse health effects are lacking^43^. A recent systematic review revealed that the pooled proportion of elderly individuals who self-medicated was 36% (95% CI 27–45%)^44^.

Importantly, a significant proportion of individuals engaging in self-medication lack adequate knowledge about its risks and safe practices, indicating the need for targeted education and awareness campaigns to promote responsible self-care behaviors and prevent adverse outcomes^45,46^. Self-medication for older adults can be dangerous even with over-the-counter medicines compared to other age groups because of age-related changes in organ functions. And it remains to be studied which medicines can be considered safe as over-the-counter medicines in older adults. The use of unsafe self-medication carries a risk to the user and the community (e.g., the spread of antimicrobial resistance). Addressing self-medication practices requires a comprehensive approach encompassing healthcare provider education, public awareness campaigns, and regulatory measures to ensure the safe and rational use of medications. Empowering individuals with accurate information about the potential risks and benefits of self-medication, along with promoting strategies for prudent medication use, can help mitigate the adverse consequences associated with inappropriate self-care practices. Moreover, healthcare providers play a crucial role in guiding older adults toward informed decisions regarding self-medication, accenting the importance of open communication and patient education within the healthcare system^47^.

This study addresses both polypharmacy and self-medication practices among older adults, offering a holistic view of medication usage in this demographic population from six different geographical regions of India. This ensures the generalizability of the results across urban community settings in India. The identification of risk factors could help in the development and testing of specific interventions to reduce unsafe medication usage among older adults. However, this study has certain limitations. First, the sample size was overall powered but not powered for individual cities for any meaningful comparisons across cities. Similarly, the sample size was not calculated to study the risk factors associated with polypharmacy and self-medication. Second, our operational definition of polypharmacy excluded nonoral medicinal formulations and nonsolid formulations, probably leading to the underestimation of the burden of polypharmacy. Third, the exclusion of individuals with severe cognitive or hearing deficits might have limited data on inappropriate medications in these individuals given the fact that cognitive deficit and inappropriate medication use are linked in older adults. In addition, no formal cognitive assessment tool was used for precise cognitive assessment. This might have limited consistency in cognitive assessment and warrants future studies using validated tools for this purpose. Fourth, the questionnaire and its translated versions regarding self-medication, adapted from pre-validated literature, underwent pilot testing to assess clarity, relevance, and cultural fit, but were not fully statistically validated for older adults. Validation studies that specifically focus on older adults are required in this regard. Finally, interviewer bias and response bias could not be fully eliminated. Despite these limitations, this study provides a snapshot of the burden and factors associated with unsafe medication usage among older adults in various Indian urban communities. If medication practices improve, treatment complications and related costs can decrease, and the quality of life of older adults can improve.

## Conclusion

This study, which was conducted across six Indian cities, revealed important findings regarding medication usage and self-medication practices among community-dwelling older adults. The prevalence of polypharmacy and unsafe self-medication was high in this population. The common associated factors were three or more comorbidities and recent hospitalization. Among those who self-medicated, a high proportion lacked knowledge of uninformed or unsafe self-medication, did not comprehend the associated risks, and reported unsafe self-medication practices. This problem needs to be addressed by raising awareness and establishing services for medication reconciliation, review, and feedback to improve medication safety and management in older adults.

## Data Availability

The datasets generated during and/or analyzed during the current study are available from the corresponding author upon reasonable request.
